# *trans*-Dichloridobis[(pyridin-4-yl)boronic acid-κ*N*]palladium(II) dimethyl sulfoxide disolvate

**DOI:** 10.1107/S1600536811010713

**Published:** 2011-04-07

**Authors:** Adam Duong, James D. Wuest, Thierry Maris

**Affiliations:** a Département de Chimie, Université de Montréal, 2900 Boulevard Edouard-Montpetit, Montréal, Québec, Canada H3C 3J7

## Abstract

In the title compound, [PdCl_2_(C_5_H_6_BNO_2_)_2_]·2C_2_H_6_OS, the Pd^II^ ion is located on an inversion centre and is four-coordinated in a *trans* square-planar geometry by two chloride ions and two (pyridin-4-yl)boronic acid ligands. The Pd—N and Pd—Cl distances are 2.023 (2) and 2.2977 (7) Å, respectively, and the average N—Pd—Cl angle is 90°. The dimethyl sulfoxide solvent mol­ecules play a key role in the crystal structure by bridging the complex mol­ecules *via* O—H⋯O hydrogen bonds, forming tapes running along the *b* axis. C—H⋯O inter­actions also contribute to the cohesion of the crystal.

## Related literature

For other Pd^II^ complexes with chloride and pyridine ligands, see: Qin *et al.* (2002 ▶); Viossat *et al.* (1993 ▶); Zordan & Brammer (2006 ▶).
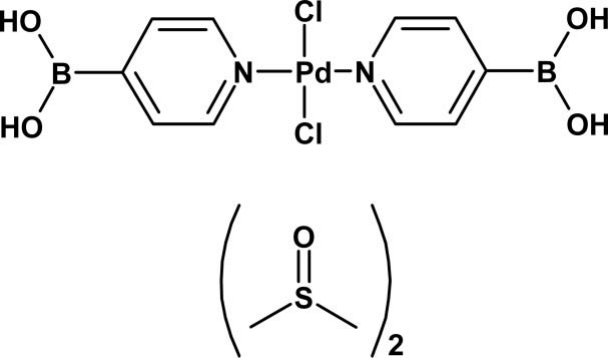


## Experimental

### 

#### Crystal data

[PdCl_2_(C_5_H_6_BNO_2_)_2_]·2C_2_H_6_OS
*M* *_r_* = 579.39
Triclinic, 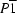

*a* = 6.2629 (4) Å
*b* = 8.1515 (5) Å
*c* = 11.7761 (7) Å
α = 80.687 (3)°
β = 82.248 (3)°
γ = 77.456 (3)°
*V* = 576.00 (6) Å^3^
*Z* = 1
Cu *K*α radiation
μ = 10.62 mm^−1^
*T* = 150 K
0.12 × 0.09 × 0.08 mm

#### Data collection

Bruker SMART 6000 diffractometer
Absorption correction: multi-scan (*SADABS*; Sheldrick, 2004 ▶) *T*_min_ = 0.252, *T*_max_ = 0.428
6942 measured reflections
2135 independent reflections
2031 reflections with *I* > 2σ(*I*)
*R*_int_ = 0.041

#### Refinement

*R*[*F* ^2^ > 2σ(*F* ^2^)] = 0.031
*wR*(*F* ^2^) = 0.082
*S* = 1.07
2135 reflections
136 parameters
H-atom parameters constrained
Δρ_max_ = 0.68 e Å^−3^
Δρ_min_ = −0.87 e Å^−3^

### 

Data collection: *SMART* (Bruker, 2003 ▶); cell refinement: *SAINT* (Bruker, 2003 ▶); data reduction: *SAINT*; program(s) used to solve structure: *SHELXS97* (Sheldrick, 2008 ▶); program(s) used to refine structure: *SHELXL97* (Sheldrick, 2008 ▶); molecular graphics: *SHELXTL* (Sheldrick, 2008 ▶) and *Materials Studio* (Accelrys, 2002 ▶); software used to prepare material for publication: *UdMX* (Maris, 2004 ▶) and *publCIF* (Westrip, 2010 ▶).

## Supplementary Material

Crystal structure: contains datablocks I, global. DOI: 10.1107/S1600536811010713/kp2316sup1.cif

Structure factors: contains datablocks I. DOI: 10.1107/S1600536811010713/kp2316Isup2.hkl

Additional supplementary materials:  crystallographic information; 3D view; checkCIF report

## Figures and Tables

**Table 1:** Hydrogen-bond geometry (Å, °)

*D*—H⋯*A*	*D*—H	H⋯*A*	*D*⋯*A*	*D*—H⋯*A*
O8—H8⋯O10^i^	0.84	1.94	2.750 (3)	163
O9—H9⋯O10	0.84	1.94	2.745 (3)	160
C5—H5⋯O10	0.95	2.51	3.253 (3)	135
C12—H12*A*⋯O8^ii^	0.98	2.54	3.506 (4)	169
C12—H12*B*⋯O9^iii^	0.98	2.53	3.372 (4)	144

Symmetry codes: (i) 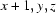; (ii) 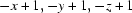; (iii) 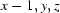.

**Fig. 1.**
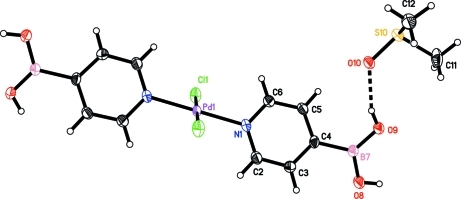
The molecular structure of the title compound with atom labels and 50% probability displacement ellipsoids for non-hydrogen atoms. Hydrogen atoms are drawn as a sphere of arbitrary radius. The unlabelled part is related by the symmetry operation -x, -y, 2 - z.

**Fig. 2.**
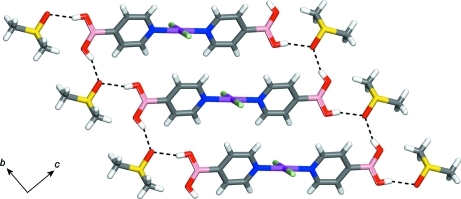
Partial view of the packing of the title compound, viewed down the a axis, showing one layer of molecules connected by O—H···O hydrogen bonds (dashed lines) involving solvent molecules of DMSO.

## supplementary crystallographic information

### Comment

The title compound was isolated as an air-stable yellow-orange solid. Each Pd^II^ centre lies on a crystallographic inversion centre in a square-planar environment. The chloride and (pyridin-4-yl)boronic acid ligands adopt a *trans* arrangement due to the molecular symmetry *C*_i_; N—Pd—Cl angles are about 90° (Fig. 1). The bond lengths expected for Pd—N and Pd—Cl (2.023 (2) Å and 2.2977 (7) Å, respectively) are similar to those observed in *trans*-dichloridobis(pyridine)Pd^II^ (Viossat *et al.*, 1993).

In the crystal structure, the solvent molecules of DMSO are linked by the boronic acid group of the complexes *via* O—H···O hydrogen bonds (average distance 2.747 (3) Å) to form tapes (Fig. 2, Table 2). The tapes are further connected to create layers by C—H···π interactions (distance C11—H11···*Cg*1 = 3.815 (4) Å where *Cg*1 is the centroid of the pyridine ring). Cohesion of the crystals also arises in part from C—H···O interactions involving one methyl moiety of DMSO molecules and oxygen atoms of the boronic acid unit (average C···O distance 3.439 (4) Å).

### Experimental

A suspension of PdCl_2_ (36 mg, 0.20 mmol) and (pyridin-4-yl)boronic acid (50 mg, 0.41 mmol) in MeCN (20 mL) was stirred for 16 h. The resulting mixture was filtered, and the solid was washed thoroughly with MeCN and then dried under vacuum before being purified by crystallization. Crystals of the title complex were grown by slow evaporation from a solution of the solid in DMSO.

### Refinement

All H-atoms were placed in calculated positions (C—H 0.95 - 0.98 Å) and were included in the refinement in the riding model approximation with U(H) set to 1.2Ueq (C) for aromatic H and 1.5Ueq (C) for methylene H. Hydroxyl H atoms were first located after a difference Fourier map calculation then refined in the riding model approximation using the AFIX 81 instruction from the *SHELX* program suite (Sheldrick, 2008), with O—H 0.84 Å and U(H) set to 1.2Ueq(O).

### Crystal data

**Table:**

[PdCl_2_(C_5_H_6_BNO_2_)_2_]·2C_2_H_6_OS	*Z* = 1
*M**_r_* = 579.39	*F*(000) = 292
Triclinic, *P*1	*D*_x_ = 1.670 Mg m^−^^3^
Hall symbol: -P 1	Cu *K*α radiation, λ = 1.54178 Å
*a* = 6.2629 (4) Å	Cell parameters from 4582 reflections
*b* = 8.1515 (5) Å	θ = 3.8–72.2°
*c* = 11.7761 (7) Å	µ = 10.62 mm^−^^1^
α = 80.687 (3)°	*T* = 150 K
β = 82.248 (3)°	Block, yellow
γ = 77.456 (3)°	0.12 × 0.09 × 0.08 mm
*V* = 576.00 (6) Å^3^	

### Data collection

**Table:**

Bruker SMART 6000 diffractometer	2135 independent reflections
Radiation source: Rotating Anode	2031 reflections with *I* > 2σ(*I*)
Montel 200 optics	*R*_int_ = 0.041
Detector resolution: 5.5 pixels mm^-1^	θ_max_ = 72.2°, θ_min_ = 3.8°
ω scans	*h* = −7→7
Absorption correction: multi-scan (*SADABS*; Sheldrick, 2004)	*k* = −10→9
*T*_min_ = 0.252, *T*_max_ = 0.428	*l* = −14→14
6942 measured reflections	

### Refinement

**Table:**

Refinement on *F*^2^	Secondary atom site location: difference Fourier map
Least-squares matrix: full	Hydrogen site location: inferred from neighbouring sites
*R*[*F*^2^ > 2σ(*F*^2^)] = 0.031	H-atom parameters constrained
*wR*(*F*^2^) = 0.082	*w* = 1/[σ^2^(*F*_o_^2^) + (0.0545*P*)^2^ + 0.0938*P*] where *P* = (*F*_o_^2^ + 2*F*_c_^2^)/3
*S* = 1.07	(Δ/σ)_max_ < 0.001
2135 reflections	Δρ_max_ = 0.68 e Å^−^^3^
136 parameters	Δρ_min_ = −0.87 e Å^−^^3^
0 restraints	Extinction correction: *SHELXL97* (Sheldrick, 2008), Fc^*^=kFc[1+0.001xFc^2^λ^3^/sin(2θ)]^-1/4^
Primary atom site location: structure-invariant direct methods	Extinction coefficient: 0.0025 (5)

### Special details

**Table:**

Experimental. X-ray crystallographic data for I were collected from a single-crystal sample, which was mounted on a loop fiber. Data were collected using a Bruker Platform diffractometer, equipped with a Bruker *SMART* 4 K Charged-Coupled Device (CCD) Area Detector using the program *APEX2* and a Nonius FR591 rotating anode equiped with Montel 200 optics The crystal-to-detector distance was 5.0 cm, and the data collection was carried out in 512 *x* 512 pixel mode. The initial unit-cell parameters were determined by a least-squares fit of the angular setting of strong reflections, collected by a 10.0 degree scan in 33 frames over four different parts of the reciprocal space (132 frames total). One complete sphere of data was collected to better than 0.80 Å resolution.
Geometry. All e.s.d.'s (except the e.s.d. in the dihedral angle between two l.s. planes) were estimated using the full covariance matrix. The cell e.s.d.'s were taken into account individually in the estimation of e.s.d.'s in distances, angles and torsion angles; correlations between e.s.d.'s in cell parameters were only used when they are defined by crystal symmetry. An approximate (isotropic) treatment of cell e.s.d.'s was used for estimating e.s.d.'s involving l.s. planes.
Refinement. Refinement of *F*^2^ against ALL reflections. The weighted *R*-factor *wR* and goodness of fit *S* are based on *F*^2^, conventional *R*-factors *R* are based on *F*, with *F* set to zero for negative *F*^2^. The threshold expression of *F*^2^ > σ(*F*^2^) is used only for calculating *R*-factors(gt) *etc*. and is not relevant to the choice of reflections for refinement. *R*-factors based on *F*^2^ are statistically about twice as large as those based on *F*, and *R*- factors based on ALL data will be even larger.

### Fractional atomic coordinates and isotropic or equivalent isotropic displacement parameters (Å^2^)

**Table:**

	*x*	*y*	*z*	*U*_iso_*/*U*_eq_	
Pd1	0.0000	0.0000	1.0000	0.01847 (14)	
Cl1	0.11789 (13)	−0.28133 (9)	0.97775 (6)	0.02895 (19)	
N1	0.2223 (4)	0.0656 (3)	0.86854 (18)	0.0200 (5)	
C2	0.3867 (5)	0.1387 (4)	0.8834 (2)	0.0236 (6)	
H2	0.4017	0.1599	0.9586	0.028*	
C3	0.5340 (5)	0.1837 (4)	0.7926 (2)	0.0233 (6)	
H3	0.6538	0.2292	0.8067	0.028*	
C4	0.5097 (5)	0.1633 (4)	0.6795 (2)	0.0203 (6)	
C5	0.3368 (5)	0.0875 (4)	0.6668 (2)	0.0217 (6)	
H5	0.3141	0.0695	0.5920	0.026*	
C6	0.1981 (5)	0.0384 (4)	0.7615 (2)	0.0223 (6)	
H6	0.0835	−0.0156	0.7511	0.027*	
B7	0.6709 (6)	0.2213 (5)	0.5709 (3)	0.0233 (7)	
O8	0.8706 (4)	0.2330 (3)	0.59305 (17)	0.0353 (6)	
H8	0.9476	0.2547	0.5305	0.042*	
O9	0.6102 (4)	0.2582 (3)	0.46159 (16)	0.0296 (5)	
H9	0.4748	0.2611	0.4638	0.036*	
O10	0.1939 (4)	0.2593 (3)	0.41211 (16)	0.0306 (5)	
S10	0.13090 (13)	0.28540 (10)	0.28929 (6)	0.02519 (19)	
C11	0.3559 (6)	0.3547 (5)	0.1998 (3)	0.0399 (9)	
H11A	0.4844	0.2613	0.2002	0.060*	
H11B	0.3909	0.4503	0.2294	0.060*	
H11C	0.3164	0.3905	0.1205	0.060*	
C12	−0.0641 (5)	0.4798 (4)	0.2747 (3)	0.0318 (7)	
H12A	−0.0058	0.5685	0.3002	0.048*	
H12B	−0.2015	0.4657	0.3223	0.048*	
H12C	−0.0925	0.5125	0.1935	0.048*	

### Atomic displacement parameters (Å^2^)

**Table:**

	*U*^11^	*U*^22^	*U*^33^	*U*^12^	*U*^13^	*U*^23^
Pd1	0.01890 (19)	0.0231 (2)	0.01229 (16)	−0.00496 (12)	0.00089 (10)	−0.00004 (11)
Cl1	0.0351 (4)	0.0237 (4)	0.0244 (3)	−0.0038 (3)	0.0060 (3)	−0.0028 (3)
N1	0.0230 (13)	0.0214 (12)	0.0139 (10)	−0.0032 (10)	−0.0004 (9)	−0.0006 (9)
C2	0.0244 (16)	0.0282 (16)	0.0172 (12)	−0.0038 (13)	−0.0012 (11)	−0.0028 (11)
C3	0.0199 (15)	0.0288 (16)	0.0215 (12)	−0.0057 (13)	−0.0036 (11)	−0.0016 (11)
C4	0.0164 (14)	0.0251 (15)	0.0165 (11)	−0.0021 (12)	0.0020 (10)	−0.0002 (10)
C5	0.0232 (15)	0.0258 (15)	0.0157 (11)	−0.0036 (12)	−0.0027 (10)	−0.0029 (10)
C6	0.0230 (15)	0.0238 (15)	0.0195 (12)	−0.0045 (12)	−0.0038 (11)	−0.0006 (11)
B7	0.0205 (17)	0.0310 (18)	0.0180 (13)	−0.0067 (14)	0.0005 (12)	−0.0026 (12)
O8	0.0205 (12)	0.0661 (17)	0.0194 (9)	−0.0150 (12)	−0.0018 (8)	0.0021 (10)
O9	0.0208 (11)	0.0519 (15)	0.0173 (9)	−0.0140 (10)	0.0004 (8)	−0.0007 (9)
O10	0.0199 (11)	0.0520 (15)	0.0180 (9)	−0.0087 (10)	−0.0045 (8)	0.0051 (9)
S10	0.0271 (4)	0.0280 (4)	0.0205 (3)	−0.0054 (3)	−0.0063 (3)	−0.0003 (3)
C11	0.0300 (19)	0.051 (2)	0.0259 (15)	0.0031 (17)	0.0088 (13)	0.0087 (15)
C12	0.0251 (17)	0.0374 (19)	0.0289 (14)	−0.0008 (14)	−0.0010 (12)	−0.0011 (13)

### Geometric parameters (Å, °)

**Table:**

Pd1—N1^i^	2.023 (2)	C6—H6	0.9500
Pd1—N1	2.023 (2)	B7—O8	1.338 (4)
Pd1—Cl1^i^	2.2977 (7)	B7—O9	1.360 (4)
Pd1—Cl1	2.2977 (7)	O8—H8	0.8400
N1—C2	1.340 (4)	O9—H9	0.8400
N1—C6	1.348 (3)	O10—S10	1.5201 (19)
C2—C3	1.372 (4)	S10—C12	1.778 (3)
C2—H2	0.9500	S10—C11	1.780 (3)
C3—C4	1.401 (4)	C11—H11a	0.9800
C3—H3	0.9500	C11—H11b	0.9800
C4—C5	1.393 (4)	C11—H11c	0.9800
C4—B7	1.594 (4)	C12—H12a	0.9800
C5—C6	1.380 (4)	C12—H12b	0.9800
C5—H5	0.9500	C12—H12c	0.9800
			
N1^i^—Pd1—N1	180.0	N1—C6—H6	119.4
N1^i^—Pd1—Cl1^i^	90.64 (7)	C5—C6—H6	119.4
N1—Pd1—Cl1^i^	89.36 (7)	O8—B7—O9	121.3 (3)
N1^i^—Pd1—Cl1	89.36 (7)	O8—B7—C4	116.3 (3)
N1—Pd1—Cl1	90.64 (7)	O9—B7—C4	122.4 (3)
Cl1^i^—Pd1—Cl1	180.0	B7—O8—H8	109.5
C2—N1—C6	119.3 (2)	B7—O9—H9	109.5
C2—N1—Pd1	122.71 (17)	O10—S10—C12	106.02 (14)
C6—N1—Pd1	117.96 (19)	O10—S10—C11	105.38 (15)
N1—C2—C3	121.6 (2)	C12—S10—C11	98.27 (17)
N1—C2—H2	119.2	S10—C11—H11A	109.5
C3—C2—H2	119.2	S10—C11—H11B	109.5
C2—C3—C4	120.8 (3)	H11A—C11—H11B	109.5
C2—C3—H3	119.6	S10—C11—H11C	109.5
C4—C3—H3	119.6	H11A—C11—H11C	109.5
C5—C4—C3	116.2 (2)	H11B—C11—H11C	109.5
C5—C4—B7	121.4 (2)	S10—C12—H12A	109.5
C3—C4—B7	122.4 (3)	S10—C12—H12B	109.5
C6—C5—C4	120.8 (2)	H12A—C12—H12B	109.5
C6—C5—H5	119.6	S10—C12—H12C	109.5
C4—C5—H5	119.6	H12A—C12—H12C	109.5
N1—C6—C5	121.2 (3)	H12B—C12—H12C	109.5
			
Cl1^i^—Pd1—N1—C2	63.4 (2)	C3—C4—C5—C6	0.7 (4)
Cl1—Pd1—N1—C2	−116.6 (2)	B7—C4—C5—C6	−179.8 (3)
Cl1^i^—Pd1—N1—C6	−114.6 (2)	C2—N1—C6—C5	−1.3 (5)
Cl1—Pd1—N1—C6	65.4 (2)	Pd1—N1—C6—C5	176.8 (2)
C6—N1—C2—C3	−1.3 (5)	C4—C5—C6—N1	1.6 (5)
Pd1—N1—C2—C3	−179.3 (2)	C5—C4—B7—O8	−156.2 (3)
N1—C2—C3—C4	3.7 (5)	C3—C4—B7—O8	23.2 (5)
C2—C3—C4—C5	−3.3 (5)	C5—C4—B7—O9	24.3 (5)
C2—C3—C4—B7	177.3 (3)	C3—C4—B7—O9	−156.3 (3)

Symmetry codes: (i) −*x*, −*y*, −*z*+2.

### Hydrogen-bond geometry (Å, °)

**Table:**

*D*—H···*A*	*D*—H	H···*A*	*D*···*A*	*D*—H···*A*
O8—H8···O10^ii^	0.84	1.94	2.750 (3)	163
O9—H9···O10	0.84	1.94	2.745 (3)	160
C5—H5···O10	0.95	2.51	3.253 (3)	135
C12—H12A···O8^iii^	0.98	2.54	3.506 (4)	169
C12—H12B···O9^iv^	0.98	2.53	3.372 (4)	144

Symmetry codes: (ii) *x*+1, *y*, *z*; (iii) −*x*+1, −*y*+1, −*z*+1; (iv) *x*−1, *y*, *z*.
